# Resident Medical Officers' Department

**Published:** 1907-12-21

**Authors:** 


### RESIDENT MEDICAL OFFICERS' DEPARTMENT.

### "INFECTIOUS DISEASES IN GENERAL HOSPITALS."

Sir,—The article on the above which appeared in The Hospital of November 16, page 180, dealt with the subject from a general point of view only, and therefore it was not stated in detail how a patient should be moved. Surely it would be an exhibition of lamentable ignorance on the part of a qualified medical man (be he junior or senior) not to know the law of the land regarding such cases. Infectious diseases and how to deal with them are included as part of the medical student's curriculum, and so the subject is one on which no one can justifiably plead ignorance.

I am, etc.,
The Writer of the Article.

